# Synthesis and Characterization of Covalent Triazine Framework CTF-1@Polysulfone Mixed Matrix Membranes and Their Gas Separation Studies

**DOI:** 10.3389/fchem.2019.00693

**Published:** 2019-10-23

**Authors:** Subarna Dey, Stefanie Bügel, Sara Sorribas, Alexander Nuhnen, Asamanjoy Bhunia, Joaquín Coronas, Christoph Janiak

**Affiliations:** ^1^Institut für Anorganische Chemie und Strukturchemie, Heinrich-Heine-Universität Düsseldorf, Düsseldorf, Germany; ^2^Chemical and Environmental Engineering Department and Instituto de Nanociencia de Aragon, Instituto de Ciencia de Materiales de Aragón (ICMA), Universidad de Zaragoza-CSIC, Zaragoza, Spain

**Keywords:** covalent triazine framework (CTF), polysulfone (PSF), mixed-matrix membrane (MMM), gas selectivity, free fractional volume

## Abstract

Covalent triazine framework CTF-1 and polysulfone (PSF) are used to form mixed-matrix membranes (MMMs) with 8, 16, and 24 wt% of the porous filler material CTF-1. Studies on permeability and selectivity are carried out concerning the gases O_2_, N_2_, CO_2_, and CH_4_. CO_2_ permeability of the synthesized MMMs increases by 5.4 Barrer in comparison to the pure PSF membrane. The selectivity remains unchanged for O_2_/N_2_ and CO_2_/CH_4_ but was found to be increased for CO_2_/N_2_. Further, comparisons to theoretical models for permeability prediction yield a permeability for CTF-1 which is about six times higher than the permeability of PSF. The inverse of the sum of the free fractional volumes (FFV) of the polymer and the filler correlate linearly to the logarithm of the permeabilities of the gases which conversely indicates that the porosity of the filler contributes to the gas transport through the membrane.

## Introduction

During the last decades membrane-based separation technology has experienced a major expansion in the gas separation industry due to advantages like low operating costs, ease of operation, minimum energy requirement, and environmental friendliness. Currently membrane-based technology is used in the chemical and petrochemical industry, for natural gas purification, hydrogen separation, nitrogen recovery, and olefin/paraffin separation (Koros and Fleming, 1993; Strathmann, 2001; Baker, 2002; Zhang et al., 2008). Polymeric membranes have been studied widely for their low costs, high processability, and good intrinsic transport properties. However, pure polymer membranes face a reciprocal trade-off relationship between permeability and selectivity (Shimekit et al., 2011). Inorganic membranes, in spite of having outstanding separation properties, good thermal, mechanical and chemical stability, suffer from high production costs, lack of processability, difficulties in large-scale production, and brittleness (Dong et al., 2013). As an alternative to polymer and inorganic membranes, mixed matrix membranes (MMMs) have attracted major attention due to their low costs, high permeabilities, and possibly selectivities above the Robeson upper-bound limit (Dong et al., 2013). A typical MMM contains a bulk continuous polymer phase and a dispersed inorganic particle phase. Polymers that are generally used to fabricate MMMs include polysulfone, polyarylates, polycarbonates, poly(arylethers), poly(arylketones), and polyimides (Tanh Jeazet et al., 2012). Porous materials that are generally incorporated to fabricate MMMs are carbon molecular sieves, zeolites, mesoporous materials, activated carbons, carbon nanotubes, and metal organic frameworks (MOFs) (Buonomenna et al., 2012; Tanh Jeazet et al., 2012; Bastani et al., 2013; Dong et al., 2013). In recent years porous organic polymers (POPs) or covalent organic frameworks (COFs) have also been explored to fabricate such membranes (Dechnik et al., 2017).

A subcategory of POPs/COFs are nitrogen-rich covalent triazine frameworks (CTFs). CTFs were first developed by Kuhn et al. by a polymerization reaction of aromatic di- or trinitrile building blocks under ionothermal conditions at 400–700°C using an excess of ZnCl_2_. The latter acts as a Lewis acid catalyst and solvent (porogen) for the polymerization reaction (Kuhn et al., 2008). Up to now, only few examples of CTF membranes were reported. Tang et al. reported an *in situ* fabricated neat CTF-membrane made from 4,4′-biphenyldicarbonitrile, which exhibits a high water permeability of 75600 Barrer and a water/ethanol selectivity of 101 for the dehydration of an 85 wt% ethanol aqueous solution at 45°C (Tang et al., 2015). Ying et al. developed a strategy for a graphene-oxide assisted restacking method to fabricate an ultrathin CTF-1 membrane, which showed a H_2_/CO_2_ selectivity of 22.3 (Ying et al., 2016).

High surface area, low density, excellent thermal and chemical stability with a large number of nitrogen functionalities make CTFs potential candidates for gas storage and separation (Bhunia et al., 2013; Dey et al., 2017). These facts suggested us to fabricate MMMs by using CTF-1 as a filler. The glassy polymer PSF was chosen as a matrix due to its good mechanical properties including a good film-formation behavior (Dechnik et al., 2016). The prepared MMMs (8, 16, and 24 wt% of CTF-1) were tested for O_2_/N_2_, CO_2_/N_2_, and CO_2_/CH_4_ separation.

## Materials and Methods

### Materials

All chemicals were purchased from commercial suppliers (Sigma-Aldrich, Acros Organics, and Alfa Aesar chemical company). Polysulfone (PSF) Ultrason S 6010 Natural was provided by BASF AG, Ludwigshafen, Germany. The gases O_2_, N_2_, CO_2_, and CH_4_ were supplied by Air Liquide (Germany) and used as received (purity 99.99%).

### Methods

Elemental analysis (CHN) was carried out on a PerkinElmer 2400 series 2 elemental analyzer. Thermal gravimetric analysis (TGA) was performed on a Netzsch TG 209 F3 Tarsus thermal gravimetric analyzer with a ramp rate of 5°C/min. A Bruker FT-IR Tensor 37 Spectrometer was used to obtain infrared (IR) spectra in the 4,000–550 cm^−1^ region with a 2 cm^−1^ resolution. Measurements were carried out on KBr disks. Powder X-ray diffraction (PXRD) was performed on a Bruker D2 Phaser diffractometer using Cu K_α1/α2_ radiation with λ = 1.5418 Å at 30 kV. 2θ angles in the range of 5–80° over a time of 2 h (0.01°/sec) were covered. Scanning electron microscopy (SEM) images were created by using a secondary electron (SE) detector equipped ESEM Quanta 400 FEG SEM. Sorption isotherms were obtained from a Micromeritics ASAP 2020 automatic gas sorption analyzer equipped with an oil-free vacuum pump (ultimate vacuum <10^−8^ mbar). Selectivity factors based on ideal adsorbed solution theory (IAST) were calculated using the software 3Psim version 1.1.0.7. Skeletal density was determined with a Helium pycnometer, Micromeritics AccuPyc 1330.

For determination of the permeability of the membranes, firstly the thickness of the membranes was measured on 10 different points using a micrometer screw. The gas permeation experiments were performed as described by Tanh Jeazet et al. (2016). The membrane with an area of 11.3 cm^2^ was placed into a permeation cell. First the permeate side was evacuated followed by evacuation of the feed side. The valve on the feed side was kept closed while pressurizing to approximately 3 bar for 2 h with a single gas. The line between vacuum pump and permeate side was closed followed by the adjustment of the feed pressure. The pressure on the permeate side was increased as the gas permeated from the feed side through the membrane to the permeate side. The linear rise of the pressure, recorded with an x-y printer, was used to calculate the permeability *P* in Barrer units.

Permeability is defined as the gas flow rate multiplied by the thickness of the material, divided by the area and by the pressure difference across the material:

(1) $$\begin{array}{l} Permeability\;\left(P\right)=\frac{flow\;rate\times thickness}{area\times pressure\;difference} \end{array}$$

(2) $$P(1Barrer)=10^{-10}\times \frac{cm^{3}\left(STP\right)\times cm}{cm^{2}\times s\;\times cmHg}$$

In the CGS system permeability can also be expressed as follows:

(3) $$P=\frac{g\times cm}{sec\times cm^{2}\times (dyne\times cm^{-2})}$$

The relationship between the different units is given as:

(4) $$\begin{array}{l} 1\left[\frac{g\times cm}{sec\times cm^{2}\times (dyne\times cm^{-2})}\right]\\ \;=\frac{\left(2.9882\times 10^{18}\right)}{M}\;\left[10^{-10}\times \frac{cm^{3}\left(STP\right)\times cm}{sec\times cm^{3}\times cm\;Hg}\right]\; \end{array}$$

The ideal gas selectivity was calculated from the single gas permeabilities by using the following equation:

(5) $$\begin{array}{l} \alpha_{ideal\;\left(\frac{O_{2}}{N_{2}}\right)}=\frac{P_{O_{2}}}{P_{N_{2}}} \end{array}$$

### Synthesis of CTF-1

CTF-1 has been synthesized according to the following procedure (Kuhn et al., 2008): a mixture of terephthalonitrile (1.28 g, 10 mmol) and anhydrous ZnCl_2_ (6.8 g, 50 mmol) was placed into a Pyrex ampoule under inert conditions. The ampoule was evacuated, sealed, and heated for 48 h at 400°C followed by cooling to room temperature. The black product was stirred with water for 72 h. Afterwards the product was isolated by filtration and again stirred with 200 mL of 2 mol/L aqueous HCl for 24 h. The resulting black powder was further washed with water, tetrahydrofuran (THF), acetone and dried under vacuum (yield 90 %).

### Preparation of MMMs

The MMMs were prepared with 0, 8, 16, and 24 wt% of CTF-1. The filler loadings were calculated according to the following equation (6) where the filler mass must be divided by the total mass of the composite:

(6) $$\begin{array}{l} Filler\;loading\;\left(wt\%\right)=\;\frac{m_{filler}}{m_{polymer}+\;m_{filler}}\times 100\;\% \end{array}$$

The PSF polymer (300 mg) was dissolved in chloroform (CHCl_3_) and CTF-1 was added to the polymer solution. The obtained dispersion was stirred for 1 week. Afterwards, the casting solution was treated for 30 min in an ultrasonic bath and was stirred for 30 min again. This cycle was repeated three times. Before casting, the dispersion was kept under stirring for 30 more minutes. The dispersion was cast into metal rings placed on a flat glass surface. A paper tissue covered funnel which was placed over the membrane after casting to prevent the contamination from dust particles as well as to control the evaporation rate. After solvent evaporation, the membrane was removed from the metal ring and was dried in a vacuum oven at 120°C overnight. The evaporation of CHCl_3_ from the membrane dispersion forms smooth defect/crack free films upon evaporation. The preparation of MMMs with weight percentages higher than 24 was not possible due to instability and brittleness of the resulting membranes.

## Results and Discussion

### Characterization of MMMs

**Figure 1:**
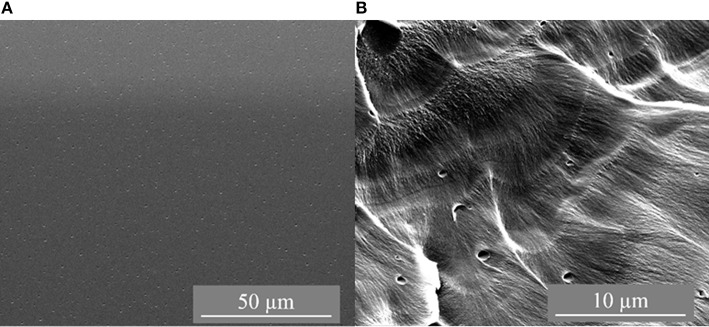
SEM images of pure PSF membrane [**(A)**: top side view; **(B)**: cross section view].

**Figure 2:**
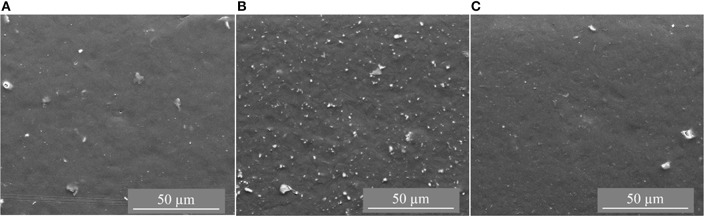
Top surface SEM images of 8 wt% **(A)**, 16 wt% **(B)** and 24 wt% **(C)** of CTF-1@PSF composite MMMs.

**Figure 3:**
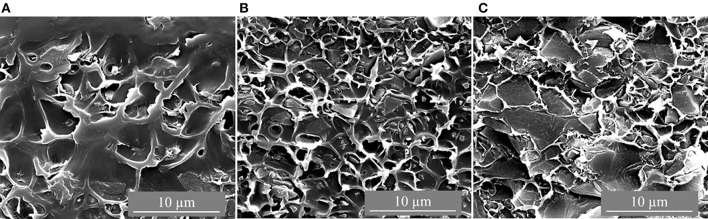
Cross-section SEM images of 8 wt% **(A)**, 16 wt% **(B)** and 24 wt% **(C)** of CTF-1@PSF MMM.

The synthesized membranes were characterized by scanning electron microscopy (SEM) with the images depicted in Figures 1–**3**. Figure 1 shows the top side and cross-section of a pure PSF flat membrane cast from CHCl_3_.

The CTF-1 composite MMMs had black appearance and were more brittle than the pure PSF membrane. Figures 2, 3 depict top surface (air side) and cross section images of 8, 16, and 24 wt% of CTF-1 composite MMMs, respectively. Figure 2 shows some, but rather few, of the CTF-1 particles at the top surfaces of the membranes. In case of sedimentation the specifically less dense CTF-1 particles should collect at the upper surface of the CH_2_Cl_2_ dispersion, which is obviously not the case. The SEM images of the membrane cross-sections (Figure 3) also indicate uniform dispersion of the CTF-1 material in the polymer matrix and no sedimentation of the CTF-1 particles was visible. The difference of the CTF-1 loading resulted in variation in the thickness of the composite membranes (Table S4). The surface images showed the incorporation of the CTF-1 particles into the polymer matrix which indicate the strong interfacial contact between PSF and CTF-1 material. The visible CTF-1 content is increased with its loading.

### Gas Permeability and Selectivity

In order to examine the gas separation performance of the pure PSF membrane and CTF-1@PSF MMMs, single-gas (O_2_, N_2_, CO_2_, and CH_4_) permeation was carried out at 25°C and 3 bar. The gas permeabilities (O_2_, N_2_, CO_2_, CH_4_) and ideal selectivity factors (O_2_/N_2_, CO_2_/CH_4_, CO_2_/N_2_) for the pure PSF and CTF-1@PSF composite membranes are provided in Table 1. For dense polymer membranes, gas separation is usually explained by a solution–diffusion mechanism (Pandey and Chauhan, 2001; Tanh Jeazet et al., 2012), which states the permeability of gas molecules through membrane as a product of diffusivity (*D*) and solubility (*S*) (Chung et al., 2007):

(7) $$\begin{array}{l} P=D\times S \end{array}$$

Diffusivity is the mobility of individual gas molecules passing through the voids between the polymeric chains of a membrane whereas gas solubility is controlled by the affinity of gas molecules toward the polymer. Addition of fillers to the polymeric membrane may affect both diffusivity and solubility which is related to physical properties of the fillers like particle size and particle agglomerations, and the polymer/particle interface morphologies, although the trend may not always be the same (Shan et al., 2016).

**Table 1:** Gas permeabilities (O_2_, N_2_, CO_2_, CH_4_) and ideal selectivity factors (O_2_/N_2_, CO_2_/CH_4_, CO_2_/N_2_) for the pure PSF and CTF-1@PSF composite membranes.

**CTF-1 load** **(wt%)**	**P** **O_2_ (Barrer)**	**P** **N_2_ (Barrer)**	**P** **CO_2_ (Barrer)**	**P** **CH_4_ (Barrer)**	**S** **O_2_/N_2_**	**S** **CO_2_/N_2_**	**S** **CO_2_/CH_4_**
0	1.6 ± 0.0	0.3 ± 0.0	7.3 ± 0.2	0.3 ± 0.0	5 ± 1	23 ± 3	21 ± 3
8	2.1 ± 0.1	0.4 ± 0.0	9.2 ± 0.6	0.4 ± 0.0	5 ± 1	23 ± 3	21 ± 3
16	2.2 ± 0.1	0.4 ± 0.0	10.7 ± 0.6	0.5 ± 0.0	5 ± 1	24 ± 3	21 ± 3
24	2.6 ± 0.2	0.5 ± 0.0	12.7 ± 0.8	0.6 ± 0.0	5 ± 1	26 ± 3	22 ± 3

*From exemplary case studies where we had the same membrane prepared several times and measured each of these membranes also several times we can generally deduce an error of 6% for permeability and 12% for selectivity through error propagation (6% + 6% for each gas permeability)*.

**Figure 4:**
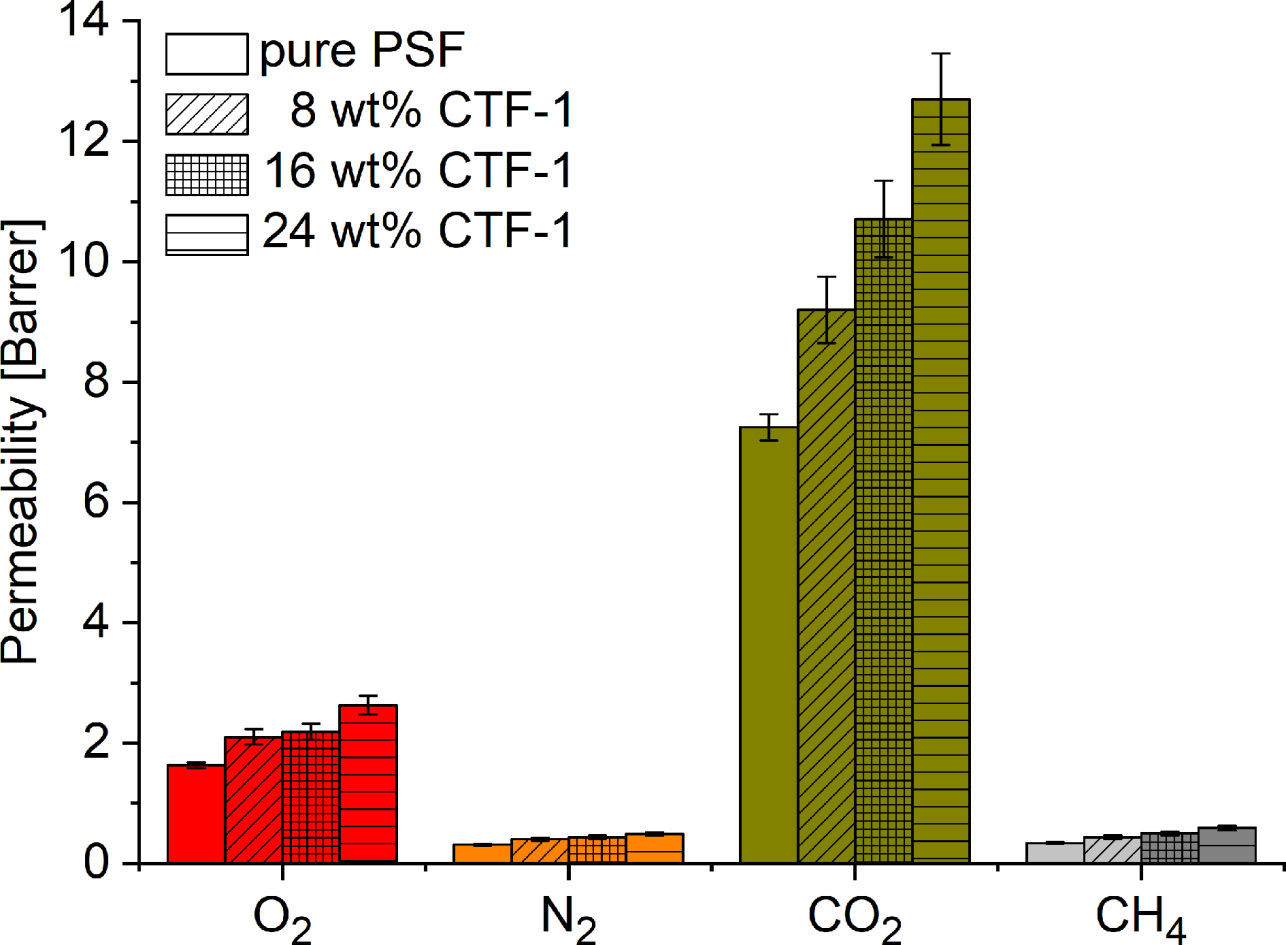
Gas permeability of 0, 8, 16, and 24 wt% of CTF-1@PSF composite MMMs for O_2_, N_2_, CO_2_, and CH_4_.

The permeability for the gases increases in proportion to the amount of CTF-1 present in the MMMs (Figure 4). The highest permeability for all the gases was found for the 24 wt% CTF-1@PSF membrane. The O_2_ permeability is increased by 63 % (from 1.6 to 2.6 Barrer), N_2_ permeability by 67 % (from 0.3 to 0.5 Barrer), CO_2_ permeability by 74 % (from 7.3 to 12.7 Barrer), and CH_4_ permeability is increased by 100 % (from 0.3 to 0.6 Barrer).

**Figure 5:**
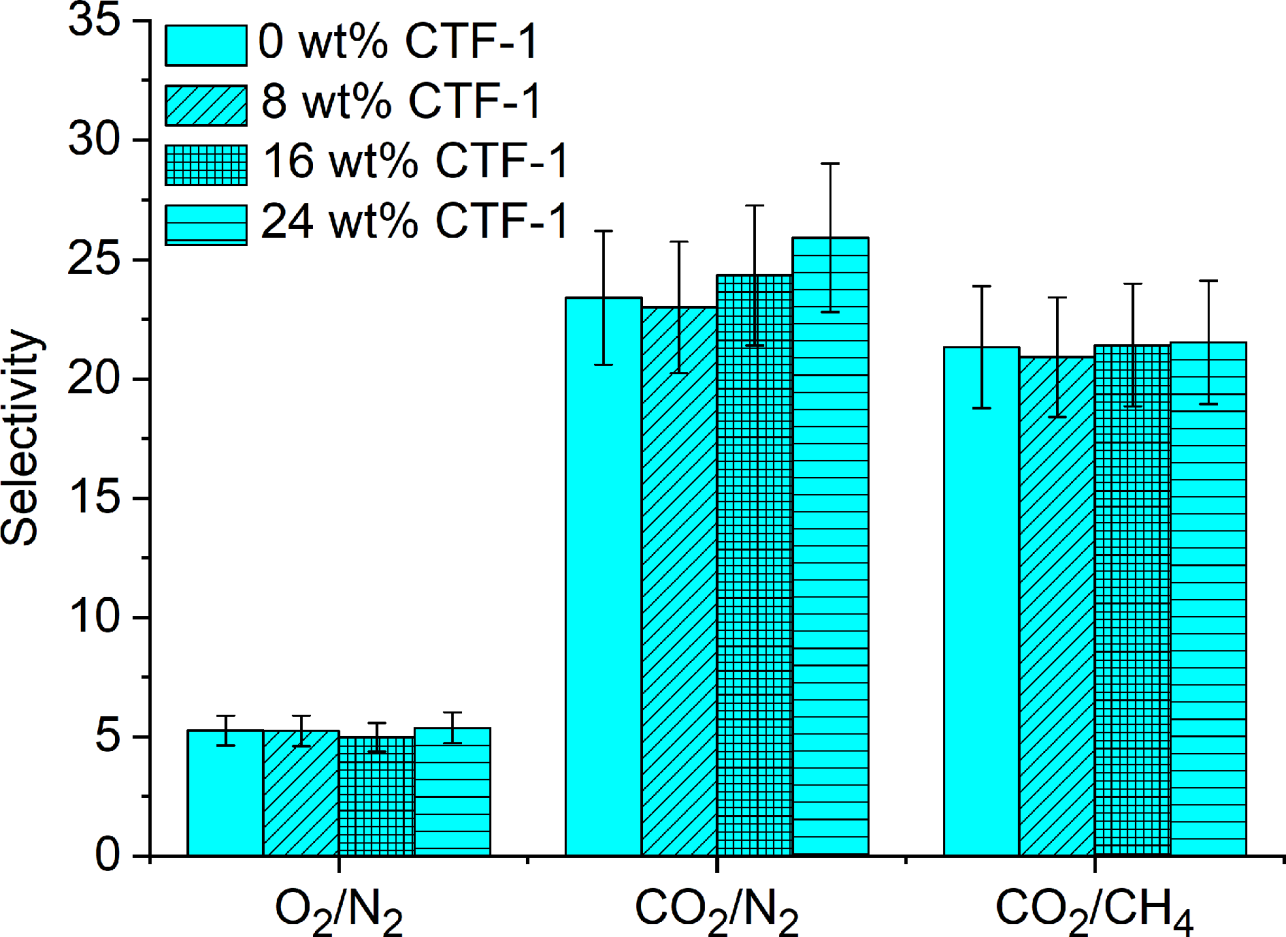
Gas selectivity of 0, 8, 16, and 24 wt% of CTF-1@PSF composite MMMs for O_2_, N_2_, CO_2_, and CH_4_.

Figure 5 shows the graphical representation of the ideal selectivity values. There is no significant improvement observed for O_2_/N_2_ and CO_2_/CH_4_ selectivity. On the other hand, CO_2_/N_2_ selectivity was found to be increased from 23 to 26. Selective CO_2_ over N_2_ adsorption of pure CTF-1 (Figure S7; Section Ideal Adsorbed Solution Theory (IAST) Calculation in the Supplementary Material) was confirmed by application of IAST (Myers and Prausnitz, 1965). The ideal selectivity factor for a binary CO_2_/N_2_ gas mixture at 1 bar pressure at 293 K is 46 and therefore explains the increase of selectivity with higher filler content in the MMMs. The higher CO_2_ permeability as well as CO_2_/N_2_ separation factors measured for the MMMs can be rationalized by the selective adsorption of CO_2_ in the nitrogen rich CTF-1 through dipole–quadrupole interactions (Li et al., 2014). When porous fillers (i.e., CTF-1) are added, the solubility may increase which is due to the higher affinity of CO_2_ toward CTF-1, as well as selective diffusivity may increase as the free volume of MMMs increases. The presence of the microporous CTF with pore diameters mainly distributed at 5, 6, and 12 Å could also exert some preferential sieving of CO_2_ (kinetic diameter of 0.33 nm) over N_2_ (0.364 nm) or CH_4_ (0.38 nm) (Li et al., 2004; Cecopieri-Gómez et al., 2007).

Generally, POPs or COFs (albeit not CTFs) were already used as filler materials in different MMMs for example with the polymers polybenzimidazole (PBI), Matrimid, or polyvinylamine (PVAm). Kang et al. incorporated two 2D COFs NUS-2 and NUS-3 as a filler in a polymer matrix (Ultem and PBI) and the membrane with 20 wt% of NUS-2 loading in PBI exhibited a H_2_/CO_2_ selectivity of 31.4 on single gas tests at high pressure which surpassed the 2008 Robeson upper bound limit (Kang et al., 2016). Shan et al. reported a MMM, using Matrimid and an azine linked COF i.e., ACOF-1, where the MMM with 16 wt% of ACOF-1 showed a CO_2_ permeability two times higher than the pure Matrimid membrane (Shan et al., 2016). A more than 3-fold elevation in CO_2_ permeability compared to the pure PVAm membrane was reported with an imine-based COF (COF-LZU1) as filler (Cao et al., 2016). Fu et al. synthesized a COF/MOF (COF-300/ZIF-8) composite membrane which gives a H_2_/CO_2_ selectivity of 13.5 in comparison to the respective COF-300 (6.0) and ZIF-8 (9.1) membranes (Fu et al., 2016). Biswal et al. introduced two hybrid membranes such as TpPa-1@PBI-BuI and TpBD@PBI-BuI (BuI = 5-t-butylisophthalic acid). Almost seven times higher permeabilities for the gases H_2_, N_2_, CO_2_, and CH_4_ could be achieved compared to the pure polymer membranes (Biswal et al., 2016). These aforementioned types of POPs have, however, low chemical and thermal stability, which limits the use for MMM based gas separation. Porous CTFs on the other hand, feature high thermal and chemical stability and often show a high CO_2_ uptake capacity and good selectivity toward CO_2_/N_2_ (Zhao et al., 2013; Hug et al., 2015).

So far, no CTF-based mixed-matrix membranes have been studied for gas permeation, to the best of our knowledge. A direct comparison can be made to a pure CTF membrane, named TFM-1 derived from 4,4′-biphenyldicarbonitrile (DCBP). The single gas CO_2_/N_2_ selectivity value of 26 for the 24 wt% CTF-1 membrane is comparable to CO_2_/N_2_ selectivity of this pure TFM-1 membrane (29 ± 2) (Zhu et al., 2012). Further, we can compare our CTF-1@PSF MMMs only to related porous organic polymer MMMs. From an N-rich Schiff based porous organic framework (SNW-1) which was constructed from melamine and di-aldehydes the derived best PSF-MMMs yielded higher CO_2_ and N_2_ gas permeabilities than CTF-1@PSF but a similar CO_2_/N_2_ selectivity of 29 in single gas measurements (Gao et al., 2014). The CO_2_ permeability of 12.7 Barrer and the selectivity of 26 in the 24 wt% CTF-1@PSF MMM is similar or even slightly better to the performance of the azobenzene-based nanoporous polymer, called Azo-COP-2, in a PSF matrix with 14.8 Barrer and a CO_2_/N_2_ selectivity of 23 (Li et al., 2019).

We have also performed mixed gas separation measurements for 400 mg PSF membranes (Table S5) for 8 and 16 wt% CTF-1@PSF MMMs. The selectivities of 8 wt% and 16 wt% of CTF-1 loading MMMs for an equimolar (50/50) gas mixture of CO_2_ and CH_4_ were found to be 40 and 42 which is higher than the single gas selectivity. Compared to single gas permeation tests, mixed gas permeation tests give higher selectivity due to the competitive adsorption and diffusion of the binary gas components in the membrane. Due to the smaller molecular size and high affinity of the CO_2_ molecule to the basic triazine unit of CTF-1, CO_2_ favorably adsorbed to the CTF-1 loaded MMMs, which reduces the diffusion of CH_4_ in the membranes due to pore blocking by adsorbed CO_2_ (Kang et al., 2016).

### Maxwell Model

A way to predict the permeability of MMMs is the application of the Maxell model. In its original form it can be used for low filler contents (φ_*d*_ up to 0.2), to exclude interactions among the filler particles (Bouma et al., 1997; Kanehashi et al., 2015). The Maxwell equation can be expressed by Equation (8):

(8) $$\begin{array}{l} P_{eff}=P_{c}\times \frac{P_{d}+2{\text{P}}_{\text{c}}-2\phi_{d}\times \left(P_{c}-P_{d}\right)}{P_{d}+2{\text{P}}_{\text{c}}+\phi_{d}\;\times \left(P_{c}-P_{d}\right)} \end{array}$$

*P*_*d*_ is given as the filler permeability and *P*_*c*_ is the permeability of the pure polymer membrane. φ_*d*_ is the volume fraction of the filler phase according to Equation (9).

(9) $$\begin{array}{l} \phi_{d}=\frac{w_{d}\;/\;\rho_{d}}{\frac{w_{c}}{\rho_{c}}\;+\;\frac{w_{d}}{\rho_{d}}} \end{array}$$

A “reduced permeation polarizability” β can be defined as given in Equation (10) (Basu et al., 2010),

(10) $$\begin{array}{l} \beta =\frac{P_{d}-P_{c}}{P_{d}+2P_{c}} \end{array}$$

and consequently Equation (8) can be simplified to Equation (11):

(11) $$\begin{array}{l} P_{eff}=P_{c}\times \frac{1+2\beta \times \phi_{d}}{1-\beta {\times \phi }_{d}} \end{array}$$

The value of β describes the difference in permeability between the continuous or polymer phase (with *P*_*c*_) and the dispersed or filler phase (with *P*_*d*_). There are three limiting cases which can be considered: The filler is much more permeable than the polymer, that is *P*_*d*_ >> *P*_*c*_ and β ≈ 1; both filler and polymer are equally permeable, that is *P*_*d*_ = *P*_*c*_ and β = 0 and the filler is non-permeable or *P*_*d*_ < < *P*_*c*_ and β ≈ −0.5 (Basu et al., 2010). In case of CTF-1 being regarded as a highly-permeable filler material (*P*_*d*_ >>*P*_*c*_), the following equation (12) is used:

(12) $$\frac{P_{eff}}{P_{c}}=\frac{1+2\phi_{d}}{1-\phi_{d}}$$

**Figure 6:**
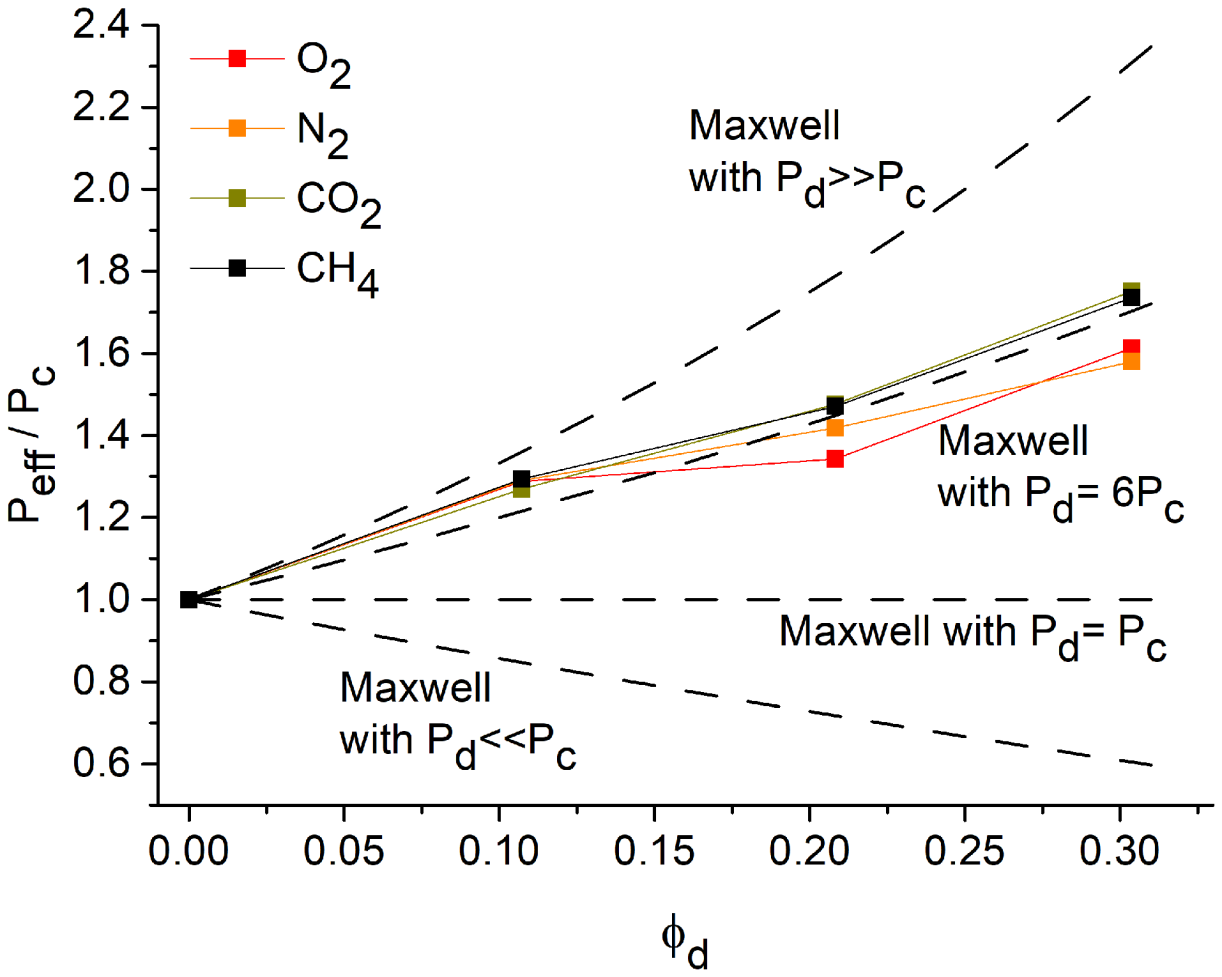
*P*_*eff*_ */P*_*c*_ vs. φ_*d*_. Measured permeabilities for the pure polymer and the polymer with 8, 16, and 24 wt% of the filler in comparison to the Maxwell model with different relations between *P*_*eff*_ and *P*_*c*_ (dashed lines).

The plot *P*_*eff*_/*P*_*c*_vs. φ_*d*_ is presented in Figure 6. The comparison with the theoretical Maxwell plot shows an agreement only in the range of very low filler contents. With a higher volume fraction of the filler, the theoretical Maxwell model predicts a higher increase in permeability. The Maxwell model describes an ideal case, which is also based on the assumption of an ideal distribution of the filler particles and the spherical shape of the filler particles (Bouma et al., 1997). The deviation from the model could be explained by the non-spherical shape of the CTF-1 particles. Another reason could be the penetration of PSF polymer chains into the pores of CTF-1 and thus a loss off free volume of the filler (Li et al., 2005).

If the pores in CTF-1 would be fully blocked and the filler thereby becomes nearly non-permeable we have the limiting case of *P*_*d*_ < < *P*_*c*_ and β ≈ – 0.5 with equation (13), with the plot of *P*_*eff*_/*P*_*c*_vs. φ_*d*_ also included in Figure 6:

(13) $$\begin{array}{l} \frac{P_{eff}}{P_{c}}=\frac{1-\phi_{d}}{1+\;0.5\phi_{d}} \end{array}$$

From Figure 6 it is evident that the measured permeability lies between the limiting case with *P*_*d*_ >> *P*_*c*_ and the case where both filler and polymer are equally permeable, that is *P*_*d*_ = *P*_*c*_ with *P*_*eff*_*/P*_*c*_ = 1. In order to therefore approximate the experimental permeability, we can assume *P*_*d*_ = 6*P*_*c*_ with β = 0.625 to give Equation (14):

(14) $$\begin{array}{l} \frac{P_{eff}}{P_{c}}=\frac{1+1.25\phi_{d}}{1-0.625\phi_{d}} \end{array}$$

The plot of Equation (14) is depicted in Figure 6 and the experimental values show good agreement with the model.

An overview of other models for the case *P*_*d*_ >> *P*_*c*_, including Bruggeman (1935), Higuchi (Higuchi and Higuchi, 1960; Shen and Lua, 2013), and Böttcher-Landauer (Hashin and Shtrikman, 1962) for *P*_*eff*_/*P*_*c*_ vs. filler fraction is given in Figure S9 (Section Other Permeability Models for 300 mg Membranes in the Supplementary Material). It is evident that the other models overestimate the permeability even more strongly than the Maxwell model for *P*_*d*_ >> *P*_*c*__._

### Fractional Free Volume (FFV)

The FFV of the filler was calculated by multiplication of the density (ρ_d_ in g/cm^3^) and the pore volume (cm^3^/g) (Thran et al., 1999). He-pycnometry combined with BET-sorption measurement was used to determine the density (ρ_d_) of CTF-1 (ρ_d_ = 0.89 g/cm^3^, dispersed phase) and the pore volume of 0.42 cm^3^/g was given by BET-sorption analysis. The density of PSF (ρ_c_ = 1.23 g/cm^3^, continuous phase) as well as the FFV_polymer_ (0.156) is used according to the literature (Thran et al., 1999; Anaya et al., 2014). In order to calculate the (total) FFV of the MMM both the FFV of the polymer and of the filler are multiplied by their respective volume fractions, φ_c_ and φ_*d*_, and summed up according to Equation (15). The volume fraction of the polymer φ_c_ was determined in analogy to Equation (9).

(15) $$\begin{array}{l} \left(total\right)FFV=FFV_{polymer}\times \phi_{c}+FFV_{filler}\times \phi_{d} \end{array}$$

**Figure 7:**
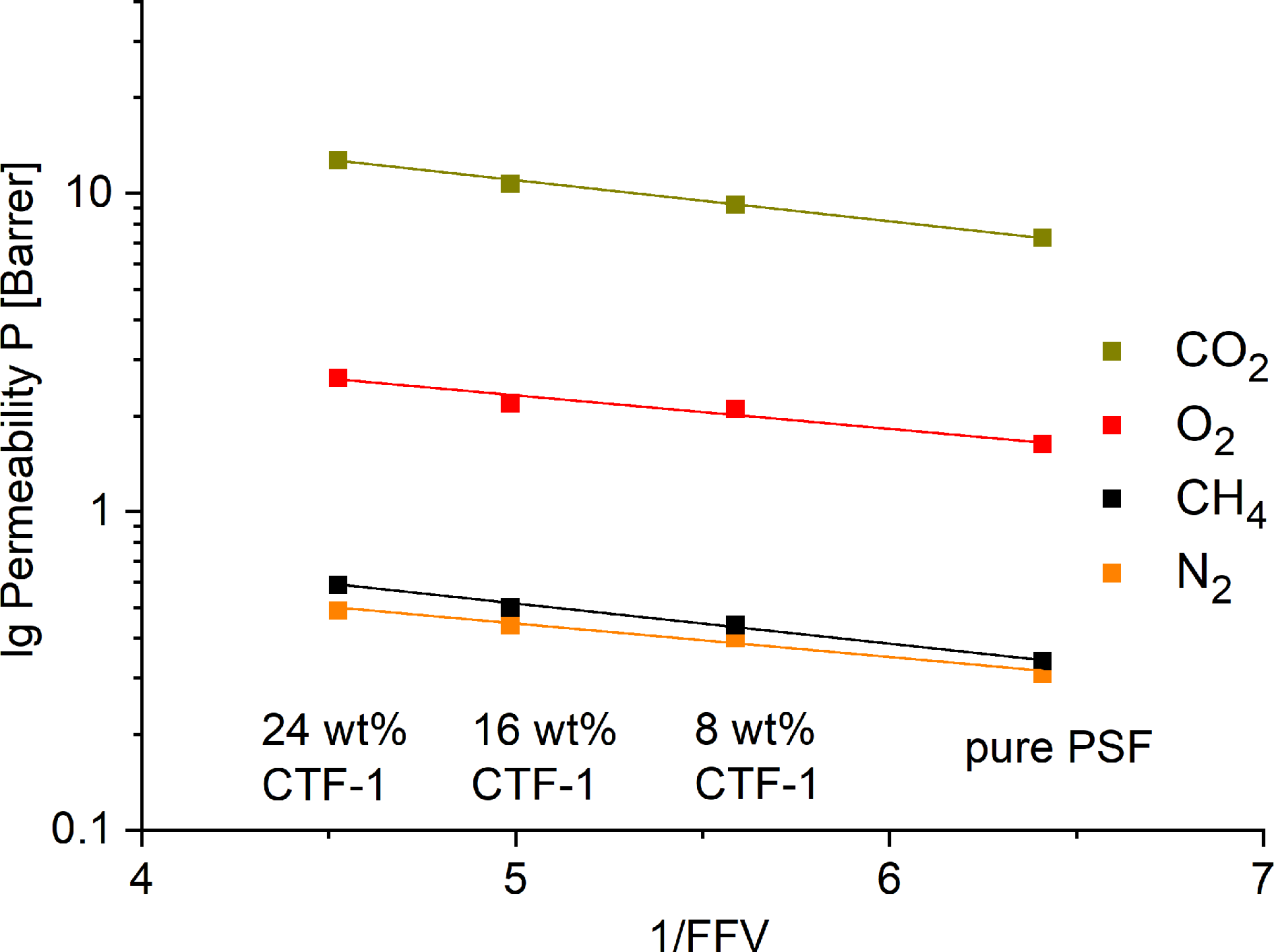
Logarithmic plot of the experimental O_2_, N_2_, CO_2_, and CH_4_ permeabilities vs. the inverse (total) FFV of the pure polymer and the polymer with 8, 16, and 24 wt% of the filler.

Figure 7 presents the logarithm of the measured gas permeabilities (lg *P*) for O_2_, N_2_, CO_2_, and CH_4_ as a function of the inverse FFV for pure PSF, 8, 16, and 24 wt% of CTF-1. The FFV for 8 wt% of the filler is 0.18, loadings of 16 and 24 wt% show values of 0.20 and 0.22. Independent from gas all plots show a linear correlation.

## Conclusion

In summary, we have successfully synthesized for the first time mixed matrix membranes containing thermally and chemically stable CTF-1 and PSF. Overall six MMMs have been casted in this study by using PSF with 8, 16, and 24 wt% CTF-1. The SEM images of the membrane cross-sections show uniform dispersion of the CTF-1 material in the polymer matrix, whereas the surface images of the MMMs indicate the strong interfacial contact between PSF and CTF-1 material. The fabricated membranes exhibit higher CO_2_ permeabilities (12.7 Barrer for 24 wt% of CTF-1 loading) than the pure PSF membrane (7.3 Barrer). For other gases there are no significant improvements in the permeability. The MMMs show higher CO_2_/N_2_ selectivity (26 for 24 wt% of CTF-1 loading) compared to pure PSF membrane (23), the selectivity increases with increasing of CTF loading. The results for higher filler contents differ from the Maxwell model for porous fillers, but a constant increase of permeability can be observed for the gases CO_2_ and CH_4_ and a modified Maxwell model was successfully applied. The increased gas permeability follows linearly the inverse of the total free fractional volume, which indicates that both free fractional volume of the polymer and the filler contribute to the permeability.

## Data Availability Statement

All datasets generated for this study are included in the article/Supplementary Material.

## Author Contributions

SD synthesized membranes and wrote half of the manuscript. SB wrote half of manuscript and applied permeability models, FFV, and drew the graphics. SS carried out mixed gas measurements. AN participated in calculations for Maxwell model and FFV. AB provided CTF-1 and related analytical information. JC was involved in mixed gas measurements and interpretation. CJ proofread and refined the manuscript.

### Conflict of Interest

The authors declare that the research was conducted in the absence of any commercial or financial relationships that could be construed as a potential conflict of interest.

## References

[B1] Anaya S. Serrano B. Herrero B. Cervera A. Baselga J. (2014). γ-alumina modification with long chain carboxylic acid surface nanocrystals for biocompatible polysulfone nanocomposites. ACS Appl. Mater. Interfaces 6, 14460–14468. 10.1021/am503744z 25065267

[B2] Baker R. W. (2002). Future directions of membrane gas separation technology. Ind. Eng. Chem. Res. 41, 1393–1411. 10.1021/ie0108088

[B3] Bastani D. Esmaeili N. Asadollahi M. (2013). Polymeric mixed matrix membranes containing zeolites as a filler for gas separation applications: a review. J. Ind. Eng. Chem. 19, 375–393. 10.1016/j.jiec.2012.09.019

[B4] Basu S. Cano-Odena A. Vankelecom I. F. J. (2010). Asymmetric Matrimid®/[Cu_3_(BTC)_2_] mixed-matrix membranes for gas separations. J. Membr. Sci. 362, 478–487. 10.1016/j.memsci.2010.07.005

[B5] Bhunia A. Vasylyeva V. Janiak C. (2013). From a supramolecular tetranitrile to a porous covalent triazine-based framework with high gas uptake capacities. Chem. Commun. 49, 3961–3963. 10.1039/c3cc41382a 23563918

[B6] Biswal B. P. Chaudhari H. D. Banerjee R. Kharul U. K. (2016). Chemically stable covalent organic framework (COF)-polybenzimidazole hybrid membranes: enhanced gas separation through pore modulation. Chem. Eur. J. 22, 4695–4699. 10.1002/chem.201504836 26865381

[B7] Bouma R. H. B. Checchetti A. Chidichimo G. Drioli E. (1997). Permeation through a heterogeneous membrane: the effect of the dispersed phase. J. Membr. Sci. 128, 141–149. 10.1016/S0376-7388(96)00303-1

[B8] Bruggeman D. A. G. (1935). Berechnung verschiedener physikalischer Konstanten von heterogenen Substanzen. 1. Dielektrizitätskonstanten und Leitfähigkeiten der Mischkörper aus isotropen Substanzen. Ann. Phys. 24, 636–679. 10.1002/andp.19354160705

[B9] Buonomenna M. G. Yave W. Golemme G. (2012). Some approaches for high performance polymer based membranes for gas separation: block copolymers, carbon molecular sieves and mixed matrix membranes, RSC Adv. 2, 10745–10773. 10.1039/c2ra20748f

[B10] Cao X. Qiao Z. Wang Z. Zhao S. Li P. Wang J. (2016). Enhanced performance of mixed matrix membrane by incorporating a highly compatible covalent organic framework into poly(vinylamine) for hydrogen purification. Int. J. Hydrogen Energy 41, 9167–9174. 10.1016/j.ijhydene.2016.01.137

[B11] Cecopieri-Gómez M. L. Palacios-Alquisira J. Domínguez J. M. (2007). On the limits of gas separation in CO_2_/CH_4_, N_2_/CH_4_ and CO_2_/N_2_ binary mixtures using polyimide membranes. J. Membr. Sci. 293, 53–65. 10.1016/j.memsci.2007.01.034

[B12] Chung T.-S. Jiang L. Y. Li Y. Kulprathipanja S. (2007). Mixed matrix membranes (MMMs) comprising organic polymers with dispersed inorganic fillers for gas separation. Prog. Polym. Sci. 32, 483–507. 10.1016/j.progpolymsci.2007.01.008

[B13] Dechnik J. Gascon J. Doonan C. J. Janiak C. Sumby C. J. (2017). Mixed-matrix membranes. Angew. Chem. Int. Ed. 56, 9292–9310. 10.1002/anie.201701109 28378379

[B14] Dechnik J. Muhlbach F. Dietrich D. Wehner T. Gutmann M. Luhmann T. (2016). Luminescent metal–organic framework mixed-matrix membranes from lanthanide metal–organic frameworks in polysulfone and matrimid. Eur. J. Inorg. Chem. 4408–4415. 10.1002/ejic.201600235

[B15] Dey S. Bhunia A. Boldog I. Janiak C. (2017). A mixed-linker approach towards improving covalent triazine-based frameworks for CO_2_ capture and separation. Micropor. Mesopor. Mater. 241, 303–315. 10.1016/j.micromeso.2016.11.033

[B16] Dong G. Li H. Chen V. (2013). Challenges and opportunities for mixed-matrix membranes for gas separation. J. Mater. Chem. A 1, 4610–4630. 10.1039/c3ta00927k

[B17] Fu J. Das S. Xing G. Ben T. Valtchev V. Qiu S. (2016). Fabrication of COF-MOF composite membranes and their highly selective separation of H_2_/CO_2_. J. Am. Chem. Soc. 138, 7673–7680. 10.1021/jacs.6b03348 27225027

[B18] Gao X. Zou X. Ma H. Meng S. Zhu G. (2014). Highly selective and permeable porous organic framework membrane for CO_2_ capture. Adv. Mater. 26, 3644–3648. 10.1002/adma.201400020 24648116

[B19] Hashin Z. Shtrikman A. (1962). Variational approach to the theory of the effective magnetic permeability of multiphase materials. J. Appl. Phys. 33, 3125–3131. 10.1063/1.1728579

[B20] Higuchi W. I. Higuchi T. (1960). Theoretical analysis of diffusional movement through heterogeneous barriers. J. Am. Pharm. Assoc. Sci. 49, 598–606. 10.1002/jps.3030490910

[B21] Hug S. Stegbauer L. Oh H. Hirscher M. Lotsch B. V. (2015). Nitrogen-rich covalent triazine frameworks as high-performance platforms for selective carbon capture and storage. Chem. Mater. 27, 8001–8010. 10.1021/acs.chemmater.5b03330

[B22] Kanehashi S. Chen G. Q. Scholes C. A. Ozcelik B. Hua C. Ciddor L. (2015). Enhancing gas permeability in mixed matrix membranes through tuning the nanoparticle properties. J. Membr. Sci. 482, 49–55. 10.1016/j.memsci.2015.01.046

[B23] Kang Z. Peng Y. Qian Y. Yuan D. Addicoat M. A. Heine T. (2016). Mixed matrix membranes (MMMs) comprising exfoliated 2D covalent organic frameworks (COFs) for efficient CO_2_ separation. Chem. Mater. 28, 1277–1285. 10.1021/acs.chemmater.5b02902

[B24] Koros W. J. Fleming G. K. (1993). Membrane-based gas separation. J. Membr. Sci. 83, 1–80. 10.1016/0376-7388(93)80013-N

[B25] Kuhn P. Antonietti M. Thomas A. (2008). Porous, covalent triazine-based frameworks prepared by ionothermal synthesis. Angew. Chem. Int. Ed. 47, 3450–3453. 10.1002/anie.200705710 18330878

[B26] Li S. Falconer J. L. Noble R. D. (2004). SAPO-34 membranes for CO_2_/CH_4_ separation, J. Membr. Sci. 241, 121–135. 10.1016/j.memsci.2004.04.027

[B27] Li S. Prasetya N. Ladewig B. P. (2019). Investigation of Azo-COP-2 as a photoresponsive low-energy CO_2_ adsorbent and porous filler in mixed matrix membranes for CO_2_/N_2_ separation. Ind. Eng. Chem. Res. 58, 9959–9969. 10.1021/acs.iecr.9b00762

[B28] Li Y. Chung T.-S. Cao C. Kulprathipanja S. (2005). The effects of polymer chain rigidification, zeolite pore size and pore blockage on polyethersulfone (PES)-zeolite A mixed matrix membranes. J. Membr. Sci. 260, 45–55. 10.1016/j.memsci.2005.03.019

[B29] Li Z. Feng X. Zou Y. Zhang Y. Xia H. Liu X.. (2014). A 2D azine-linked covalent organic framework for gas storage applications. Chem. Commun. 50, 13825–13828. 10.1039/C4CC05665E 25253410

[B30] Myers A. L. Prausnitz J. M. (1965). Thermodynamics of mixed-gas adsorption. AICHE J. 11, 121–127. 10.1002/aic.690110125

[B31] Pandey P. Chauhan R. S. (2001). Membranes for gas separation. Prog. Polym. Sci. 26, 853–893. 10.1016/S0079-6700(01)00009-0

[B32] Shan M. Seoane B. Rozhko E. Dikhtiarenko A. Clet G. Kapteijn F. (2016). Azine-linked covalent organic framework (COF)-based mixed-matrix membranes for CO_2_/CH_4_ separation. Chem. Eur. J. 22, 14467–14470. 10.1002/chem.201602999 27535016

[B33] Shen Y. Lua A. I. (2013). Theoretical and experimental studies on the gas transport properties of mixed matrix membranes based on polyvinylidene fluoride. AICHE J. 59, 4715–4726. 10.1002/aic.14186

[B34] Shimekit B. Mukhtar H. Murugesan T. (2011). Prediction of the relative permeability of gases in mixed matrix membranes. J. Membr. Sci. 373, 152–159. 10.1016/j.memsci.2011.02.038

[B35] Strathmann H. (2001). Membrane separation processes: current relevance and future opportunities. AICHE J. 47, 1077–1087. 10.1002/aic.690470514

[B36] Tang Y. P. Wang H. Chung T. S. (2015). Towards high water permeability in triazine-framework-based microporous membranes for dehydration of ethanol. ChemSusChem 8, 138–147. 10.1002/cssc.201402816 25394279

[B37] Tanh Jeazet H. B. Sorribas S. Román-Marín J. M. Zornoza B. Téllez C. Coronas J. (2016). Increased selectivity in CO_2_/CH_4_ separation with mixed-matrix membranes of polysulfone and mixed-MOFs MIL-101(Cr) and ZIF-8. Eur. J. Inorg. Chem. 27, 4363–4367. 10.1002/ejic.201600190

[B38] Tanh Jeazet H. B. Staudt C. Janiak C. (2012). Metal–organic frameworks in mixed-matrix membranes for gas separation. Dalton Trans. 41, 14003–14027. 10.1039/c2dt31550e 23070078

[B39] Thran S. Kroll G. Faubel F. (1999). Correlation between fractional free volume and diffusivity of gas molecules in glassy polymers. J. Polym. Sci. B 37, 3344–3358. 10.1002/(SICI)1099-0488(19991201)37:23<3344::AID-POLB10>3.0.CO;2-A

[B40] Ying Y. Liu D. Ma J. Tong M. Zhang W. Huang H. (2016). A GO-assisted method for the preparation of ultrathin covalent organic framework membranes for gas separation. J. Mater. Chem. A 4, 13444–13449. 10.1039/C6TA04579K

[B41] Zhang Y. Musselman I. H. Ferraris J. P. Balkus K.J J.r. (2008). Gas permeability properties of Matrimid membranes containing the metal-organic framework Cu–BPY–HFS. J. Membr. Sci. 313, 170–181. 10.1016/j.memsci.2008.01.005

[B42] Zhao Y. F. Yao K. X. Teng B. Y. Zhang T. Han Y. (2013). A perfluorinated covalent triazine-based framework for highly selective and water–tolerant CO_2_ capture. Energy Environ. Sci. 6, 3684–3692. 10.1039/c3ee42548g

[B43] Zhu X. Tian C. Mahurin S. M. Chai S.-H. Wang C. Brown S.. (2012). A Superacid-catalyzed synthesis of porous membranes based on triazine frameworks for CO_2_ separation. J. Am. Chem. Soc. 134, 10478–10484. 10.1021/ja304879c 22631446

