# Supplementary material for: Synthesis and Characterization of Covalent Triazine Framework CTF-1@Polysulfone Mixed Matrix Membranes and Their Gas Separation Studies
Source: Front Chem. 2019 Oct 23;7:693. doi: 10.3389/fchem.2019.00693 (PMC6819498; doi:10.3389/fchem.2019.00693)
Supplement: Supplementary file 1 [file Data_Sheet_1.PDF]

Supplementary Material

Supplementary Material

Synthesis and characterization of covalent triazine framework CTF-1@polysulfone mixed matrix membranes and their gas separation studies

Table of contents

S1. Characterization of CTF-1 1
S1.1. N_2 sorption and pore size distribution 3
S1.2. H_2 and CO_2 sorption 4
S1.3. Ideal adsorbed solution theory (IAST) calculation 5
S2. Membrane thickness of CTF-1@ polysulfone mixed matrix membranes 6
S3. CTF-1@polysulfone (400 mg) mixed matrix membranes 6
S3.1. Scanning electron microscopy (SEM) 6
S3.2. Mixed gas measurements 7
S4. Other permeability models for 300 mg membranes 8
S5. References 9

S1. Characterization of CTF-1

CTF-1 was used from the same batch as mentioned in the work of Bhunia et al. (Bhunia et al., 2015). The idealized structure of CTF-1 is shown in Scheme S1.

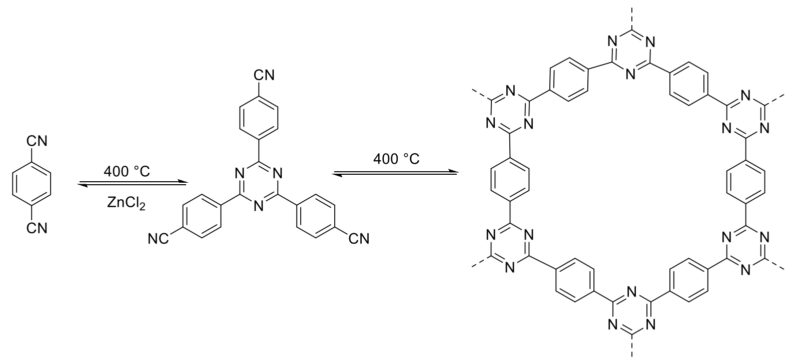

Scheme S1: Idealized structure of CTF-1 from the polymerization of terephthalonitrile.

The IR spectrum (**Fig. S1**) at 2225 cm^{-1} indicated the presence of unreacted nitrile groups in the polymer. The strong IR band around 1514 cm^{-1} is due to the C–N stretching mode of the triazine ring, whereas the band at 1352 cm^{-1} is due to in-plane stretching vibrations of the triazine ring (Hug et al., 2014). From TGA it is observed that the CTF-1 is stable up to 450 °C. The PXRD pattern (**Fig. S2**) displayed the crystalline nature of the CTF with hexagonal packing of pores (Bhunia et al., 2013). The SEM image (**Fig. S3**) showed that the material consisted particles of irregular shapes with an average particle size of about 10 µm. Elemental analysis of CTF-1 (**Table S1**) showed much lower nitrogen content which is due to nitrogen elimination during the high temperature polymerization reaction as observed by us and others (Bhunia et al., 2013). The density of CTF-1 was determined by addition of the volume of CTF-1 measured by He-pycnometry and the pore volume obtained by N_2 sorption. The mass of the sample was divided by the sum of the volumes.

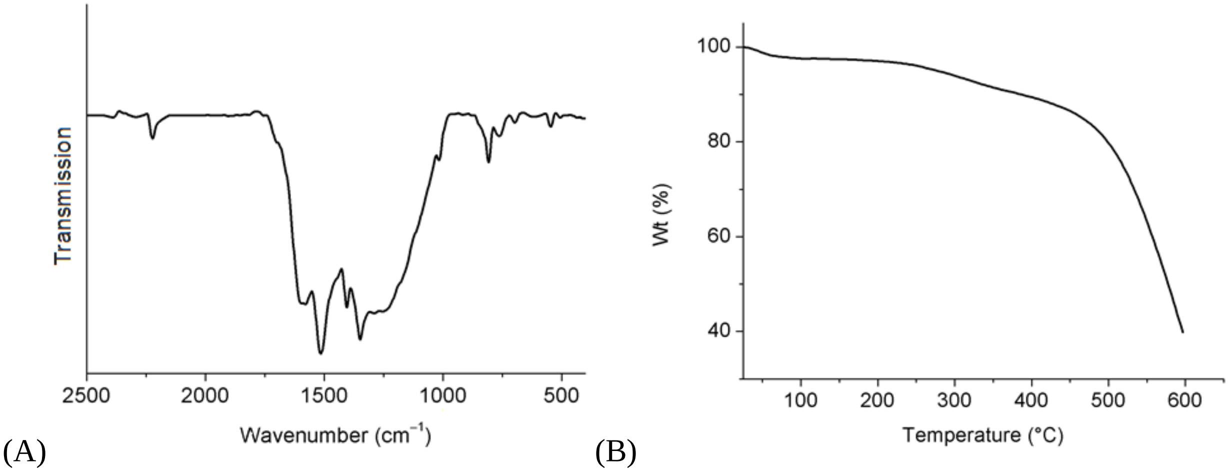

Fig. S1: FT-IR spectrum (A) and TGA data (B) for CTF-1**.**

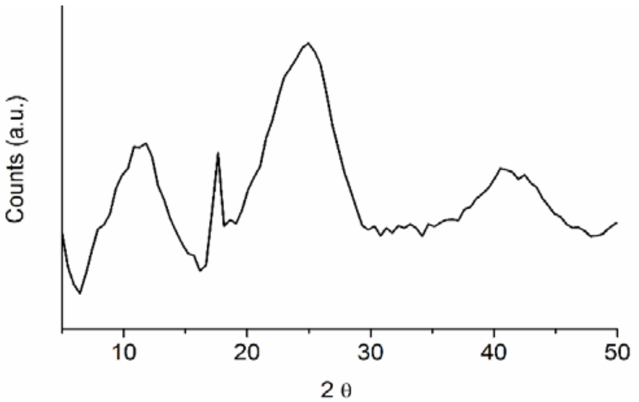

Fig. S2: Powder X-ray diffraction pattern of CTF-1.

(A) 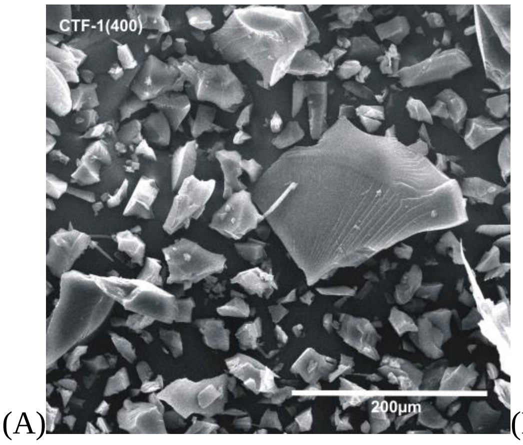

(B) 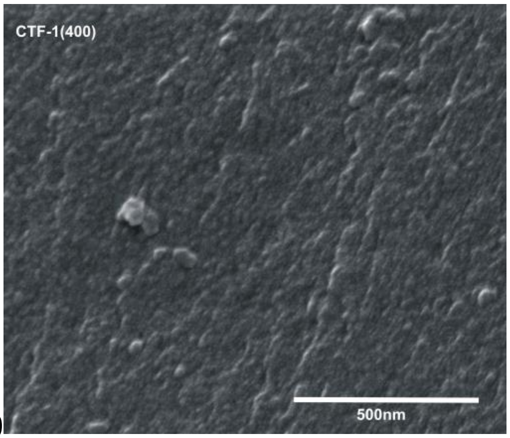

Fig. S3: SEM images of CTF-1.

Table S1: Elemental analysis of CTF-1.

Compound	Temp. (°C)	Calculated (wt%)					Found (wt%)				
		C	H	N	C/H	C/N	C	H	N	C/H	C/N
CTF-1	400	74.99	3.15	21.86	1.98	4.00	72.03	2.96	13.82	2.03	6.08

S1.1. N_2 sorption and pore size distribution

The porosity of the CTF-1 was characterized by N_2 sorption measurements as the accepted standard for surface area and pore size determination. The materials were activated by degassing at 200 °C for 24 h. The measured BET surface area for CTF-1 is 968 m^2/g. To understand the nature of porosity, non-local density functional theory (NLDFT) pore size distributions using a slit-pore model based on the N_2 adsorption isotherms were calculated. A narrow distribution of micropores centered mainly at 5, 6 and 12 Å were observed for CTF-1.

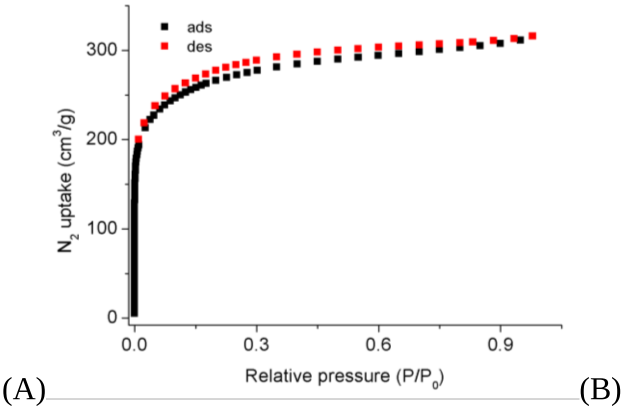

(A)
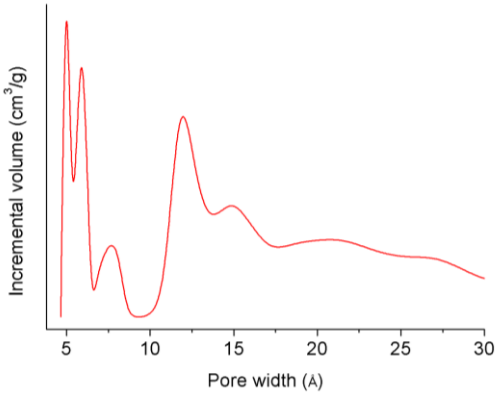

(B)

Fig. S4: Nitrogen adsorption-desorption isotherms for CTF-1 (A). NL-DFT pore size distribution curve of CTF-1 (B).

We also measured N_2 sorption at 293 K for CTF-1 (**Fig. S5**). The maximal N_2 uptake was 4.6 cm^3/g at a pressure of 724 mmHg.

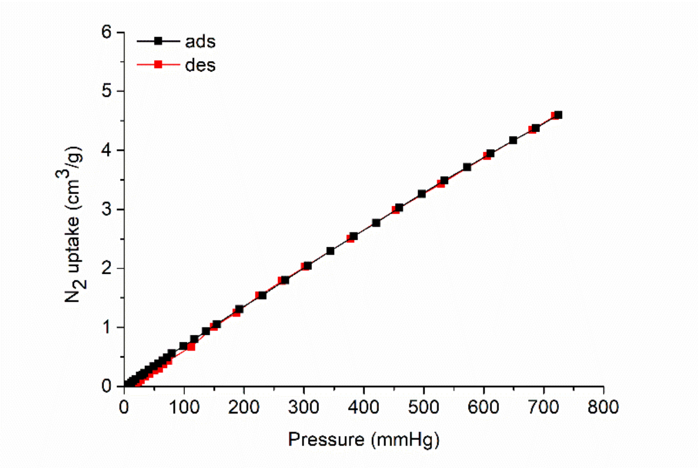

Fig. S5: Nitrogen adsorption-desorption isotherms for CTF-1 at 293 K.

S1.2. H_2 and CO_2 sorption

We investigated the adsorption of other gases, such as H_2 and CO_2 at low pressure (**Table S2**, **Fig. S6**). CTF-1 adsorbed 1.2 wt% (136 cm^3/g) H_2 at 77 K and 1 bar. The CO_2 uptake capacities of CTF-1 were measured at two different temperatures at 1 bar. The volume of CO_2 adsorption on CTF-1 at 273 K and 293 K were 72 and 49 cm^3/g, respectively.

Table S2: Gas uptake of CTF-1.

Compound	S_{BET} (m^2/g)[a]	S_{Lang} (m^2/g)	H_2 uptake at 77 K (cm^3/g)[b]	CO_2 uptake at 273 K (cm^3/g)[b]	CO_2 uptake at 293 K (cm^3/g)[b]
CTF-1	968	1181	136	72.4	49.2

[a]Calculated BET surface area over the pressure range 0.01–0.05 P/P_0. [b]Gas uptake at 1 bar.

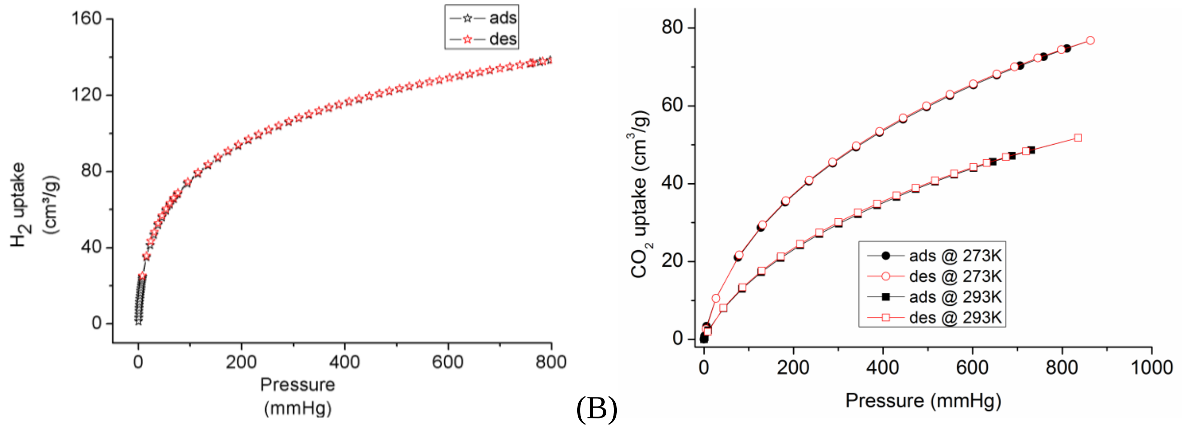

(A) (B)

Fig. S6: H_2 sorption at 77 K (A) and CO_2 sorption at 273 K and 293 K (B) for CTF-1.

S1.3. Ideal adsorbed solution theory (IAST) calculation

CO_2 and N_2 sorption measurements were carried out at 293 K (**Fig. S5** and **Fig. S6B**). The CO_2/N_2 selectivity at 293 K (**Fig. S7**) of CTF-1 was calculated using dual-site Langmuir (DSLAI) fitted isotherm data based on following equation (1):

$$q_{eq} = q_{max1} \times \frac{K_1 \times p}{1+K_1 \times p} + q_{max2} \times \frac{K_2 \times p}{1+K_2 \times p} \qquad (1)$$

The selectivities were calculated using following equation (2):

$$S = \frac{x_1/y_1}{x_2/y_2} \qquad (2)$$

with: x_i - absorbed gas amount (mmol/g); y_i – mole fraction

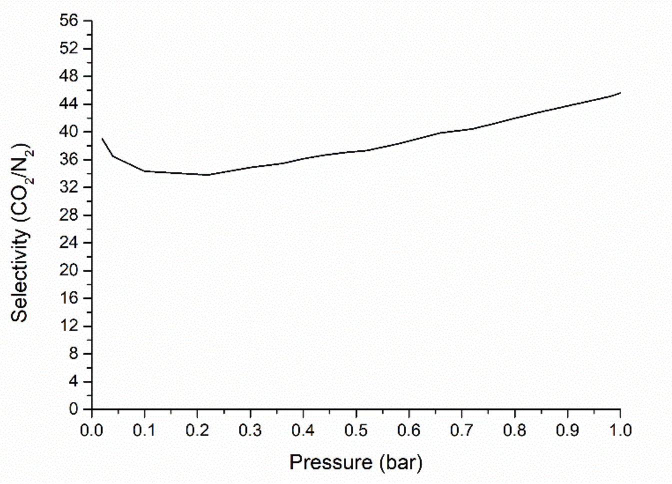

Fig. S7: IAST selectivity of CTF-1 in dependence of the pressure for a binary (50:50; v:v) mixture of the gases CO_2/N_2 at 293 K.

Table S3: Parameters for DSLAI fitting.

Gas	Temp. [K]	Model	R^2	Affinity const. 1 [1/bar]	Max. loading 1 [mmol/g]	Affinity const. 2 [1/bar]	Max. loading 2 [mmol/g]
CO_2	293	DSLAI	0.999	11.290	0.533	0.594	4.108
N_2	293	DSLAI	0.992	2.221	0.011	0.197	1.192

S2. Membrane thickness of CTF-1@ polysulfone mixed matrix membranes

The thickness of the 300 mg PSF membranes (54 µm - 76 µm) and the thickness of the 400 mg membranes (54 µm - 72 µm) is summarized in **Table S4**.

Table S4: Thickness of pure PSF membranes and MMMs.

CTF-1 load (wt%)	Thickness (µm)	
	300 mg PSF	400 mg PSF
0	54	54
8	42	72
16	50	71
24	76	70

S3. CTF-1@ polysulfone (400 mg) mixed matrix membranes

The following membranes were synthesized analogue to the 300 mg membranes.

S3.1. Scanning electron microscopy (SEM)

(A)
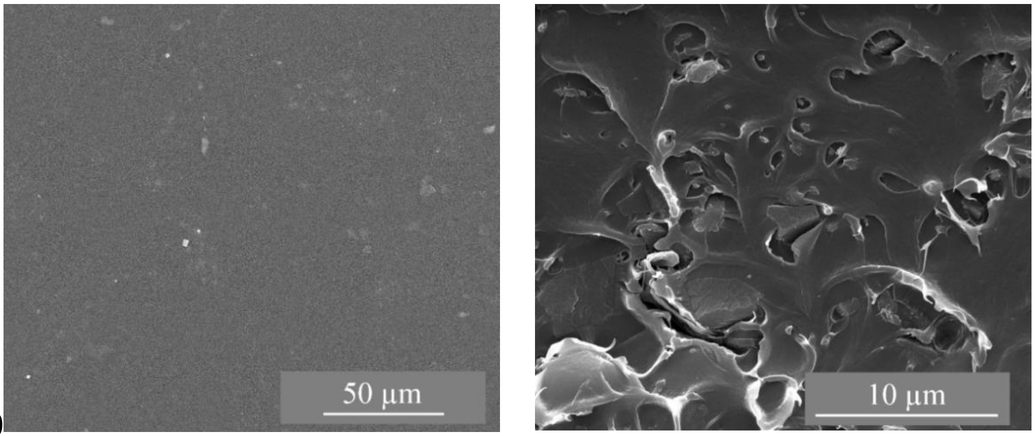

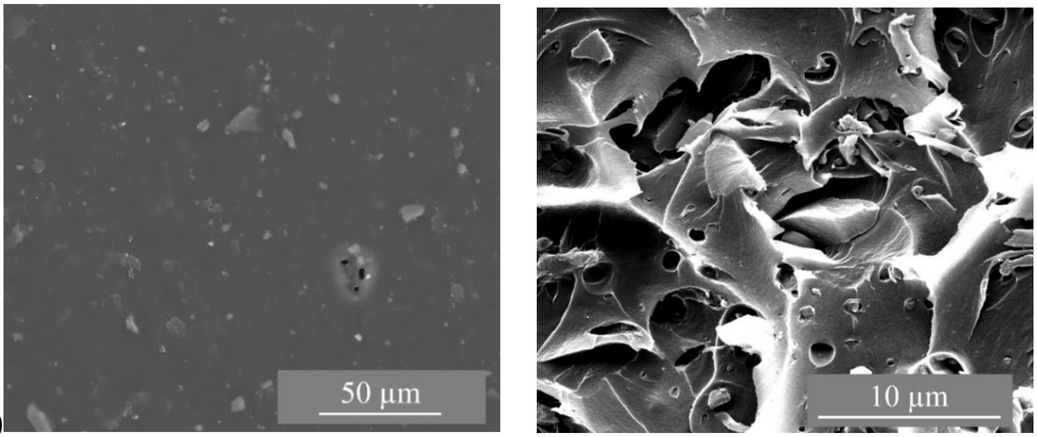

(B)

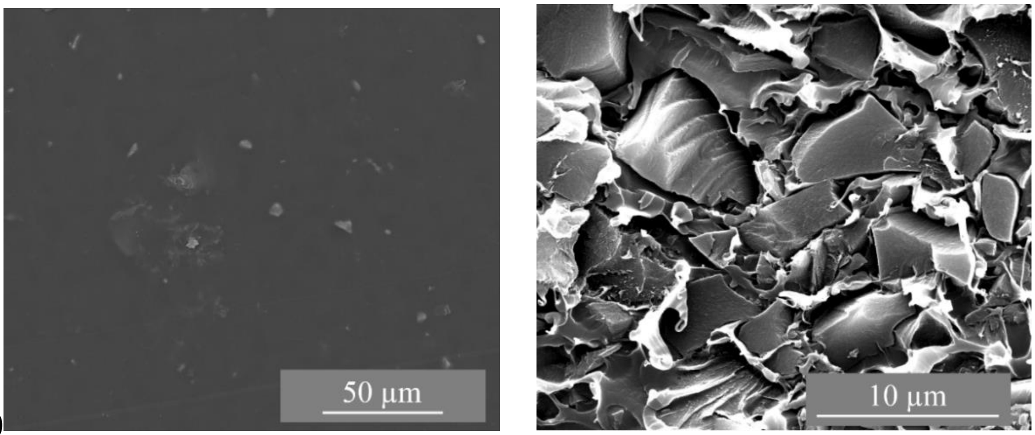

(C)

Fig. S8: Top surface SEM images (left side) and cross-section SEM images (right side) of 8 wt% (A), 16 wt% (B) and 24 wt% (C) of CTF-1@PSF (400 mg) composite MMMs.

S3.2. Mixed gas measurements

Mixed gas permeability and selectivity was determined as explained by Tanh Jeazet et al. (Tanh Jeazet et al., 2016). The membranes were placed inside a permeability module built of two stainless steel rings with a macroporous disk support (20 µm nominal pore size, Mott Corp.) gripped inside with Viton o-rings. Feed and sweep gas were provided to the membrane module by mass-flow meter controllers (Alicat Scientific). The retentate side was fed with a CO_2/CH_4 (25/25 cm^3 (STP)/min) mixture stream at ~200 kPa, while a 1 cm^3 (STP)/min mass-flow controlled stream of Ar at 110–120 kPa (slightly higher than the atmospheric pressure) swept the permeate side. An Agilent 3000A on-line gas micro-chromatograph equipped with a thermal conductivity detector (TCD) was used to analyze the gas concentrations in the outgoing stream. After the exit stream of the membrane was stabilized the permeability was obtained in Barrer (1 Barrer = $10^{-10} cm^3(STP)\cdot cm/(cm^2\cdot s\cdot cmHg)$). The real separation selectivity of the mixtures was calculated as the ratio of permeabilities. Permeation measurements were performed at 35°C controlled by a Memmert UNE 200 oven.

The CO_2 and CH_4 permeabilities as well as the CO_2/CH_4 selectivities for pure PSF and CTF-1 loadings of the MMMs with 8 wt% and 16 wt% were measured (**Table S5**). The values are shown for 400 mg polymer amount. The measurements were done at a temperature of 35 °C.

Table S5: Mixed gas separation of pure PSF and CTF-1@PSF MMMs.

Polymer amount (mg)	CTF-1 loading (wt%)	P (CO_2) (Barrer)	P (CH_4) (Barrer)	S (CO_2/CH_4)
400	0	6.1	0.2	31
400	8	7.2	0.2	40
400	16	9.3	0.2	42

S4. Other permeability models for 300 mg membranes

Besides the Maxwell model for following assumptions: $P_d >> P_c$, $P_d = P_c$, $P_d << P_c$ and $P_d = 6P_c$ (**Fig. 6** in the manuscript) the Bruggeman model (Bruggeman, 1935) was applied using following equation (3):

$$\frac{P_{eff}}{P_c} = \frac{1}{(1-\phi_d)^3} \quad (3)$$

The Higuchi model (Higuchi and Higuchi, 1960; Shen and Lua, 2013) can be calculated as in equation (4):

$$\frac{P_{eff}}{P_c} = \frac{0.22+2.78\phi_d}{0.22-0.22\phi_d} \quad (4)$$

The Böttcher-Landauer model (Hashin and Shtrikman, 1962) was simplified to the following equation (5):

$$\frac{P_{eff}}{P_c} = \frac{1}{(1-3\phi_d)} \quad (5)$$

All applied models are valid for the assumption $P_d >> P_c$. **Fig. S9** shows the graph P_{eff}/P_c versus the filler fraction ϕ_d.

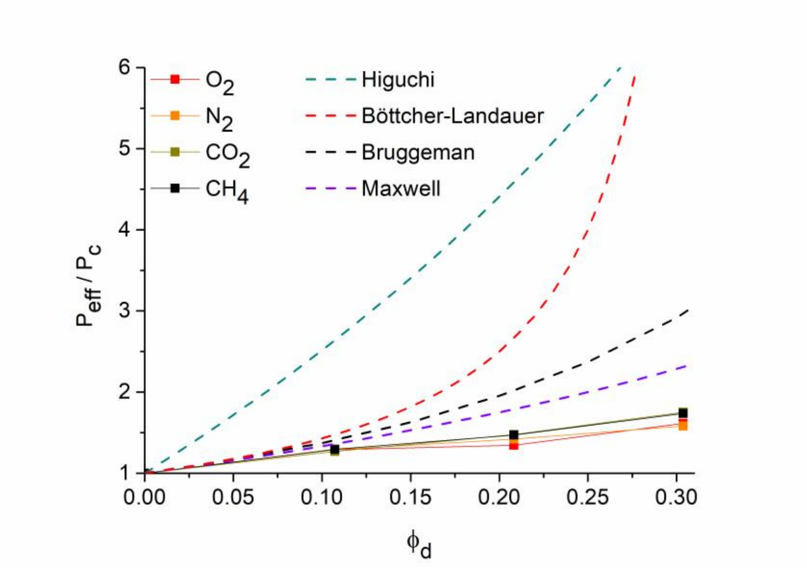

Fig. S9: P_{eff} /P_c versus ϕ_d. Measured permeabilities for the pure polymer and the polymer with 8 wt%, 16 wt% and 24 wt% of the filler CTF-1 in comparison to the Higuchi, Böttcher-Landauer, Bruggeman and Maxwell model for the assumption $P_d >> P_c$.

S5. References

Bhunia, A., Vasylyeva, V. and Janiak, C. (2013). From a supramolecular tetranitrile to a porous covalent triazine-based framework with high gas uptake capacities. *Chem. Commun.* 49, 3961−3963. doi: 10.1039/C3CC41382A

Bhunia, A., Dey, S., Bous, M., Zhang, C., von Rybinski, W. and Janiak, C. (2015). High adsorptive properties of covalent triazine-based frameworks (CTFs) for surfactants from aqueous solution. *Chem. Commun.* 51, 484−486. doi: 10.1039/C4CC06393G

Bruggeman, D.A.G. (1935). Berechnung verschiedener physikalischer Konstanten von heterogenen Substanzen. 1. Dielektrizitätskonstanten und Leitfähigkeiten der Mischkörper aus isotropen Substanzen. *Ann. Phys.* 24, 636‑679. doi: 10.1002/andp.19354160705

Hashin, Z. and Shtrikman A. (1962). Variational Approach to the Theory of the Effective Magnetic Permeability of Multiphase Materials. *J. Appl. Phys.* 33, 3125−3131. doi: 10.1063/1.1728579

Higuchi, W.I. and Higuchi T. (1960). Theoretical analysis of diffusional movement through heterogeneous barriers. *J. Am. Pharm. Assoc. Sci.* 49, 598−606. doi: 10.1002/jps.3030490910

Hug, S., Mesch, M.B., Oh, H., Popp, N., Hirscher, M and Senkerd, J. (2014). A fluorene based covalent triazine framework with high CO_2 and H_2 capture and storage capacities. *J. Mater. Chem. A* 2, 5928–5936. doi: 10.1039/C3TA15417C

Tanh Jeazet, H.B., Sorribas, S., Román-Marín, J.M., Zornoza, B., Téllez, C. and Coronas, J. (2016). Increased Selectivity in CO_2/CH_4 Separation with Mixed-Matrix Membranes of Polysulfone and Mixed-MOFs MIL-101(Cr) and ZIF-8. *Eur. J. Inorg. Chem*. 27, 4363−4367. doi: 10.1002/ejic.201600190

Shen, Y. and Lua, A.I. (2013). Theoretical and Experimental Studies on the Gas Transport Properties of Mixed Matrix Membranes Based on Polyvinylidene Fluoride *AIChE J*, 59, 4715–4726. doi: 10.1002/aic.14186
